# Interpreting and Utilising Intersubject Variability in Brain Function

**DOI:** 10.1016/j.tics.2018.03.003

**Published:** 2018-06

**Authors:** Mohamed L. Seghier, Cathy J. Price

**Affiliations:** 1 Cognitive Neuroimaging Unit, Emirates College for Advanced Education, PO Box 126662, Abu Dhabi, United Arab Emirates; 2 Wellcome Centre for Human Neuroimaging, University College London, Institute of Neurology, WC1N 3BG, London, UK

**Keywords:** neuroimaging, functional variability, brain structure, cognitive strategies, individualised predictions, covariance

## Abstract

We consider between-subject variance in brain function as data rather than noise. We describe variability as a natural output of a noisy plastic system (the brain) where each subject embodies a particular parameterisation of that system. In this context, variability becomes an opportunity to: (i) better characterise typical versus atypical brain functions; (ii) reveal the different cognitive strategies and processing networks that can sustain similar tasks; and (iii) predict recovery capacity after brain damage by taking into account both damaged and spared processing pathways. This has many ramifications for understanding individual learning preferences and explaining the wide differences in human abilities and disabilities. Understanding variability boosts the translational potential of neuroimaging findings, in particular in clinical and educational neuroscience.

## Highlights

A wealth of scientifically and clinically relevant information is hidden, and potentially invalidated, when data are averaged across subjects.

There is growing interest in using neuroimaging to explain differences in human abilities and disabilities. Progress in this endeavour requires us to treat intersubject variability as data rather than noise.

Our plastic and noisy brains intrinsically change the parameterisation of each individual’s brain, providing a rich opportunity to understand differences in brain function.

Normal variability can be used to decode different neural pathways that can sustain the same task (degeneracy).

This is of paramount importance for understanding why patients have variable outcomes after damage to seemingly similar brain regions.

## Celebrating Variability

No two human brains are identical, with variability in brain anatomy and function emerging from how each individual brain is genetically built and shaped by its intimate interaction with the environment. Most functional imaging studies discount this variability to establish how subjects typically execute a given task, with the assumption that brain activations are spatially and temporally similar across subjects. Methods for measuring intersubject variability have mainly focused on developing models of normal brain function (i.e., norms) that allow abnormality to be quantified in patient populations. This quantification of norms relies on a reductionist framework that aims to collapse the data across the subject dimension and focus on the significant common (i.e., overlapping) or mean effects (Box 1). Such over-reliance on **aggregate statistics** (see Glossary) in the pursuit of the norm can invalidate some of the conclusions drawn from group level analyses.

**Figure I:**
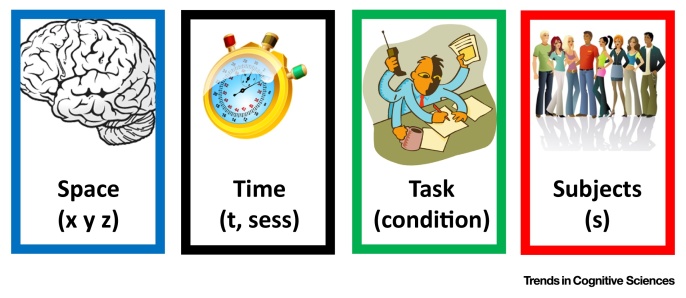
The 6D Output of a Typical Multisubject Experiment.

Box 1 **Variability along the Sixth Dimension**

A typical functional neuroimaging experiment (e.g., fMRI) requires the analysis of multidimensional data. Each experiment can be defined as a 6D dataset (Figure I): (i–iii) the three space dimensions, (iv) time, (v) an experimentally manipulated ‘task’, and (vi) ‘subject’, where the same experiment is repeated in multiple subjects. Other extra dimensions may include (vii) ‘session’ for longitudinal or repeated studies, and (viii) ‘group’ when subjects are drawn from different healthy or clinical populations. The focus here is about the sixth dimension ‘subject’.

In group studies, the information along the sixth dimension is typically treated as a ‘nuisance’ and is deliberately ‘compressed’ or ‘reduced’ to make group inferences. This approach hides other sources of variability. Different facets of variability can manifest in different dimensions, including (i) intertrial variability between events and items within the same run that might be linked to changes in strategy or learning [105]; (ii) inter-regional variability (or spatial variability) in the neurovascular coupling or BOLD sensitivity [106]; (iii) intrasubject (or intersession) variability related to the reliability and reproducibility of fMRI findings, in longitudinal or test–retest studies [91]; (iv) interindividual variability, also known as intersubject, between-subject, or across-subject variability (focus of the current review); (v) intersite variability between different scanning environments, which is sometimes a concern for large databases that include scans from different laboratories [107]; and (vi) variability in methodology: related to contextual or situational factors, for instance, differences in experimental design, acquisition sequences, and analysis methods [108].

We argue here that functional variability between subjects reflects the behaviour of the brain under parameterisation that is specific to the individual. Accordingly, the more two subjects differ in their parameterisation, the more their brain function will differ. The main structural and physiological parameters (measurable at the mesoscopic or macroscopic level) that govern such individual-specific parameterisation are: grey matter density [43]; cortical thickness [53]; morphological anatomy 109, 110; cortical layers [111]; white matter circuitry (tracts and pathways) 1, 47; myelination [44]; callosal topography [112] that influences the degree of functional lateralisation across subjects; functional connectivity [113] and its association with variability in task-related brain activity 114, 115, 116 and its divergence from structural connectivity [45]; brain oscillations and rhythms 13, 117, 118, metabolism [119] and vasculature [120]; and neurotransmitters and hormones 121, 122.

Alt-text: Box 1

The search for the mean group effect (i.e., **central tendency**), typically defined as the ultimate representative subject, implicitly treats variability that cannot be explained by any experimental manipulation as a **nuisance**, **noise,** or **measurement error**. This ignores many relevant sources of intersubject variability, including the use of different **cognitive strategies** for the same task 1, 2, 3 (Box 2), differences in learning or subjective judgment 4, 5, and the inherent normal variance in ability and capacity [6]. More critically, when meaningful information about the individual is treated as measurement error, the estimates from the group mean might not actually describe anyone well 7, 8, 9, 10, 11, 12, 13. This is why there have been many appeals, for instance, in the field of psychology, to treat between-subject variance as data rather than noise 14, 15, 16, 17, 18, 19. However, the importance of treating variance as data rather than noise has not yet been widely embraced by the neuroimaging community and most of the widely used analysis software packages only provide estimates of group effects. This is in part because the characterisation of intersubject variability requires a large number of observations from a large number of individuals, but also because of the challenge of developing methodologies for analysing, interpreting, and using between-subject variance 12, 20.

**Figure I:**
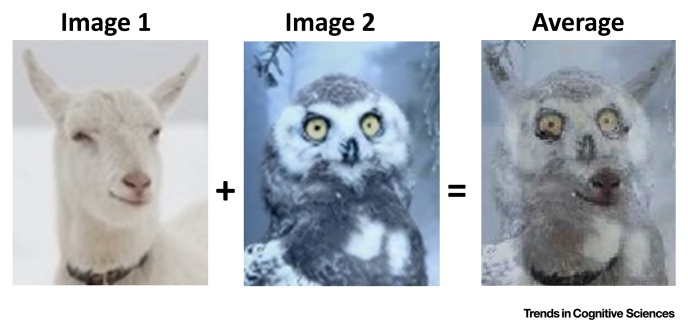
Averaging Images with Variable Features.

**Figure II:**
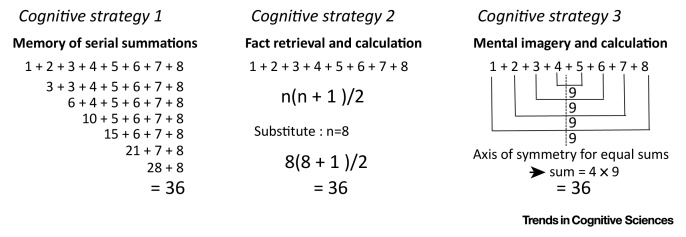
Cumulative Sums of Integers via Three Possible Strategies.

Box 2 **Intersubject Variability in Cognitive Strategy**

In typical multisubject neuroimaging studies, tasks are assumed to be performed in the same way or using a single strategy [123]. However, many tasks are unconstrained, allowing subjects to adopt their own strategy. Figure I intuitively visualises the problem of averaging individual activation maps that differ in their features [124], when subjects adopt different strategies. The resulting average in this toy example depicts a hybrid image that differs to that encoded in each of the original images. This average image contains many false negatives (where features vary between images) and false positives (where feature combinations create new features).

A hypothetical example is a task that requires subjects to hold and manipulate many numbers when performing a serial addition of successive integers. Practically, inside the scanner, the subject is shown an Arabic digit (e.g., 8) and his/her task is to verbally say the exact sum of all numbers from 1 up to the presented digit (sum of 1 to 8). If researchers have a limited knowledge of the many ways (or strategies) by which the task can be executed, they will assume that all subjects will do the task in exactly in the same way. However, there are at least three known cognitive strategies to execute this task as illustrated in Figure II.

Obviously, each strategy involves specific cognitive processes, with distinct activation patterns. This yields high between-subject variability and weak effects when the analysis sums over strategy. In contrast, paying attention to the individual pattern may help to (i) understand that different strategies do exist, and (ii) potentially predict the strategy that the individual was using. For tasks in which the strategies are known *a priori* (e.g., reading), researchers can use clever experimental manipulations to push the participant (implicitly or explicitly) towards a particular strategy. For other tasks, strategies can be inferred by looking at structure or patterns in the across-subject dimension of the group data.

Perhaps more importantly, the different types of variability in brain parameters and cognitive strategies are intimately connected. For example, slow changes in structural parameters such as grey matter (density and volume), white matter connectivity, and vasculature underpin the faster, more dynamic changes in endogenous functional connectivity and **brain rhythms**, which in turn influence task-related brain activity and behaviour 114, 115, 116, 125, 126, 127. Conversely, individual differences in cognitive strategies, cognitive styles, expectation, and decisions modulate the underlying brain structure 27, 48.

Alt-text: Box 2

In this review, we (i) describe how the brain, as a noisy **plastic** biological system, generates individual differences; (ii) review evidence of the intimate relationship between variability in brain structure and functional activation; (iii) propose that a dominant source of intersubject variability arises from **degeneracy** when the same task can be performed in multiple different ways; (iv) highlight the importance of generating explanatory models of intersubject variability when characterising and interpreting atypical activation in clinical cohorts; and (v) consider the type of methodology that is needed to investigate intersubject variability in brain activation. In brief, we treat intersubject variability as an opportunity rather than a handicap, with the ultimate aim of supporting personalised investigations of brain function.

## The Brain as a Noisy Plastic System

**Figure 1:**
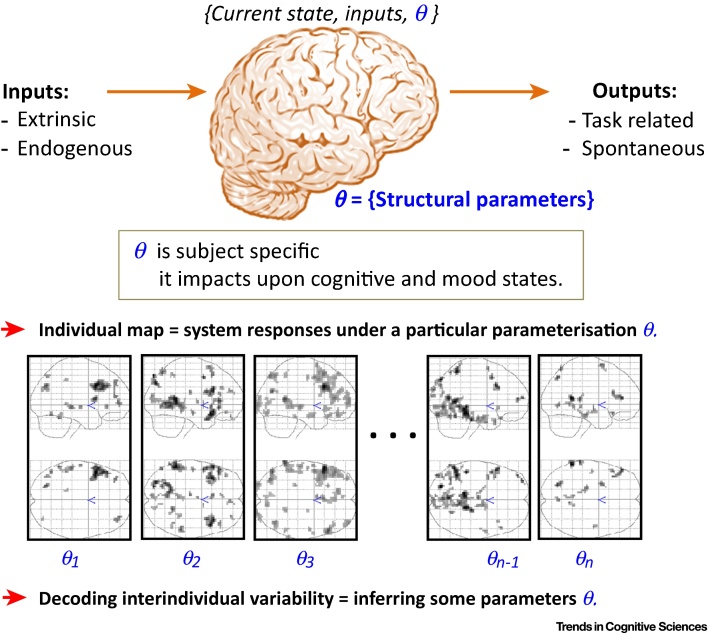
Key Figure: Individual-Specific Brain Parameterization and Variability in Brain Function

The human brain is governed by the same fundamental physical rules that posit noise in any system at non-zero temperature. Noise can convey information about microscopic processes that determine the macroscopic behaviour of the system. Indeed, many studies have described the behaviour of the human brain as a noisy **dynamical system** that generates variable responses, even in primary sensory regions 21, 22, 23, 24. The brain is also a plastic system that is subjected to continuous changes in its structure and function from the molecular to **network** levels 25, 26, 27, 28. This noisy plastic system is incessantly influenced by subject-specific endogenous (i.e., self-generated) and external (sensory) inputs, and it produces outputs at different space–time scales even during rest or sleep. Each individual brain is therefore intrinsically parameterised at microscopic, **mesoscopic**, and macroscopic levels 29, 30, 31. By studying subject-specific functional imaging responses, we can investigate how the brain operates and behaves under parameterisation that is specific to the individual (Figure 1, Key Figure), and we can ultimately extract meaningful information about that individual 32, 33, 34.

Meaningful intersubject variability in brain activation can be described as intrinsic, strategic, or contextual. Intrinsic variability arises from inherent factors rather than learned preference [35] and is therefore time invariant in the normal brain. For example, although language processing is typically a left hemisphere function, it is lateralised to the right hemisphere in ∼7% of individuals [36], and this variability has primarily been attributed to intrinsic (genetic) factors [37] that are unlikely to change over time. One way to minimise the expression of intrinsic variability is to match subjects for known demographic variables (e.g., age, gender, and handedness). In contrast to intrinsic variability, strategic (or learned) variability is constrained and shaped by prior learning. For instance, if subjects learned to execute a given task in one of many possible ways then they may adopt this initial learning strategy each time they repeat the task (Box 2). Decoding variability that is consistent within an individual, can therefore give clues to differences in **cognitive style** and preferred **cognitive strategy**. The third type of variability is contextual, which is driven, for example, by familiarity with the scanning environment and mood states that affect cooperation, motivation, habituation, awareness, and stress. This tends to change more rapidly than either intrinsic or strategic variability. Its contribution can be minimised by treating each subject with identical scanning environment and **task manipulations**.

As mentioned above, strategic variability emerges when each individual can adopt a different strategy that best matches his/her expectations and prior knowledge. The exact sources of such individual differences in strategies are not fully understood, but may include complex interactions between genetic and environmental factors. From what we currently know, there is no single gene or environmental factor that taken alone would predict exactly which strategy an individual would adopt or how an individual would learn to perform a given task. It is more likely that the interaction of genetic and environmental factors leaves its signature on the individual’s brain, continually modifying and shaping its parameterisation (Boxes 1 and 2) 38, 39, 40. These plastic changes, within a participant, contribute to more stable cross-sectional differences in brain structure that can be quantified and investigated, and may provide clues as to how genetic and environmental factors influence behaviour.

## Variability in Brain Structure and Function

There has been an abundance of studies showing how interindividual variability in brain structure is intimately related to interindividual variability in brain activation 41, 42. For instance, the degree to which grey matter density is lateralised across individuals is proportional to the degree to which language activation is left or right lateralised [43], and between-subject differences in white matter integrity are proportional to task-related brain activity [1], functional dynamics 13, 44, intrinsic functional connectivity [45], and behavioural responses 46, 47, 48. It is therefore not surprising that generative models (simulations) of brain function are improved when structural variation is accounted for 49, 50.

Variability in structure–function relationships is governed by the large number of parameters that operate in the noisy plastic brain. However, not all parameters are independent 45, 51, 52. For instance, several previous studies, reviewed in [51], have shown correlations between different structural parameters [53]. Specifically, cortical thickness and surface area in one brain region influence the cortical thickness and surface area of other structurally connected regions [54]. Structural parameters (e.g., grey matter density and white matter connectivity) also show a dependency with genetics and age, with the effect of age being consistent with what we know about developmental trajectories of structural changes [55]. Understanding these relationships [56] will ultimately help to reduce dimensionality in an informed way, with major implications for studies interested in the dynamics of the brain 57, 58, and how alterations in these relationships can cause abnormal behaviour in neurological and psychiatric conditions 59, 60, 61, 62.

## Degeneracy

An important source of normal variability in brain structure and function arises when the same task can be performed in multiple different ways. This is a type of degeneracy that is defined as ‘the ability of elements that are structurally different (e.g. brain regions, body parts, genetic codes etc.) to perform the same function or yield the same output,’ [63]. In cognitive science, a well-known example is the task of reading aloud familiar words that involves linking visual processing of text to motor processing of speech articulators, using either sublexical spelling to sound rules or whole-word recognition [64]. The degree to which each strategy and its corresponding neural system are used will depend on how an individual is taught (‘look and say’ or ‘sound it out’), their potential for learning each strategy, the degree to which they have practiced, and the proficiency they have attained [65].

At the neural level, differences in cognitive strategy will be reflected in differences in task-related brain activity because different processing types will engage different brain regions and change the way the same set of regions interact with one another. Numerous neuroimaging studies have shown significant differences in brain activation between subjects who relied upon different cognitive strategies during the execution of the same task despite similar behavioural performances 1, 4, 8, 66, 67, 68, 69, 70. These findings have implications for how multisubject activation is interpreted (Box 2).

If the structure–function relationship were the same for each individual, the set of brain regions activated could be used to predict (i.e., decode) which cognitive strategy an individual is using [19], their behavioural performance 71, 72, or their practice-induced learning 9, 73. Having learnt these associations, we could reverse the inference to predict which brain regions will be engaged when we know which strategy an individual uses. However, **functional anatomy** is not consistent across all individuals because, as discussed above, there are known sources of intrinsic intersubject variability in functional anatomy, particularly in individuals that have atypical hemispheric **lateralisation** (e.g., for language or spatial processing). These variables are only thought to account for <30% of between-subject variance 1, 68 but need to be taken into account when predicting structure from function or function from structure.

## Clinical Implications for Patients

By investigating the most common types of intersubject variability in the neural systems that support a range of functions, we can better understand and explain why outcomes or symptoms can vary from patient to patient, even when they have seemingly similar lesion sites. To illustrate, let us assume a particular cognitive task is normally performed by either a set of regions [X] or a different set of regions [Y]. If [X] is damaged, the task can still be supported by [Y], and conversely if [Y] is damaged, the task can still be supported by [X]. However, if both [X] and [Y] are damaged, a deficit ensues because there are no available ways to perform the task. Data supporting this rationale have been demonstrated with fMRI and connectivity analyses that dissociated different neural **processing pathways** for reading highly familiar object names [74] and lesion analyses that showed that damage to two pathways had a greater impact on reading than damage to one pathway had [75]. These findings support the fact that the effect of brain damage is best understood in terms of the combination of brain areas that have been damaged rather than the lesion volume or the presence or absence of damage to one particular area; for a detailed discussion see [76].

Furthermore, the distinction between intrinsic and strategic variability that we made above can help to explain interpatient differences in the speed of **recovery** and in functional **reorganisation** after brain damage 77, 78. For example, if we know that healthy subjects can effortlessly switch back and forth between different neural systems/strategies when performing the same task (i.e., strategic variability), we can predict that patients should be able to compensate rapidly and efficiently for loss of one system by switching to another prelearnt system. Alternatively, if healthy subjects show a strong preference for one neural system but can, with practice, learn to use another strategy/neural system, we would expect that the speed of recovery, following loss of the preferred system, would depend on the time taken for the patient to learn to use a nonpreferred system. Intervention in this case could focus on therapies that help the patient to use the available system. If, however, there is no evidence that healthy subjects or patients can use more than one system for a given task, then between-subject variance might be intrinsic rather than strategic, and full recovery will be slower than when multiple systems are available within subjects. This kind of information can be helpful when it comes to integrating brain plasticity and learning systems into the context of diagnosis [79], rehabilitation [80], and single-patient predictions [81].

In summary, intersubject differences in normal function provide a robust system for explaining how the brain supports recovery after focal brain damage. In this context, it is reasonable to derive the likelihood of recovery on the basis of how much variability the typical normal population shows in functional activation. Critically, however, intersubject variability needs to be assessed in a wide range of neurologically healthy and brain damaged individuals before it can be used to predict outcomes in new patients.

##  Methods for Investigating and Interpreting Intersubject Variability

Although carefully designed paradigms can be used to guide task execution with instructions to use specific strategies, the type of strategy each subject is using can only be inferred from behavioural data such as accuracy, response times, error types, learning rates, or from postscan debriefing questionnaires. When different strategies result in similar behavioural responses, statistical structure in between-subject variance in brain activation can be investigated [67], with the goal of dissociating neural systems that may each support a different (equally efficient) strategy for the same task (see hypothetical example in Box 3). If these dissociations are meaningful, they may show a relationship with independently observed variability in demographic information (age, gender, handedness, and education), out of scanner behaviour (e.g., accuracy and response times on a range of tasks that are independent of the scanner assessments), brain structure (grey matter density, regional cortical volume, and white matter tracts), or prior knowledge of the strategies used by subjects when performing the task. Such relationships between variables would typically be masked by standard averaging approaches in group analyses. The characterisation of intersubject variability can therefore complement standard group analyses and enrich the conclusions that can be drawn beyond simple mean (central) effects (see discussion in [82]).

Box 3 **Sampling and Making the Most of Intersubject Variability**

A general statistical framework for decoding unknown biological variability involves repeated sampling. In the hypothetical example illustrated here, a naïve observer wants to characterise human dexterity with no prior knowledge. He/she does this by recruiting as many humans as possible and asking them to move a ping-pong ball over a table. After observing many subjects, he/she concludes that subjects (i) always use one of their hands but (ii) differ in which hand they use (right or left). By observing many subjects successfully executing the same task, our naïve observer discovers the different ways that human dexterity is typically supported, with no prior anatomical models about the function of each body part. However, our observer cannot rule out other strategies for this task unless atypical subjects who lost both their hands are recruited. If the task can only be performed by the hands, those without hands will not be able to perform the task. If other strategies are possible, the observer will identify what these are (e.g., using the elbows or feet). The results can then be used to predict how a new individual will perform the task, with or without hands.

Modelling normal and atypical intersubject variability can help researchers to make the most of intersubject variability in brain function. This has potentially many implications in different fields including: (i) neurological and psychiatric studies to help explain variance in lesion–symptoms associations 75, 76, improve predictions about the likelihood of recovery at the individual patient level 81, 128, and enhance diagnostic power in identifying subjects at risk [129]; (ii) educational neuroscience to understand typical and atypical developmental processes [78], and to develop teaching methods from knowledge of intersubject variability in brain function, particularly in learning capacity, memory, motivation, and attention [130]; (iii) brain (mind) reading [131] and in decoding cognitive states [132]; (iv) genetic studies of normal behaviour and disease risk [133]; for instance, by correlating molecular genetic variations with interindividual differences in brain functions [134]; (v) databasing and data mining of heterogeneous populations 128, 133; and (vi) neuroergonomics that uses knowledge of brain function and human performance to design technologies and work environments for more efficient operation [135].

Modelling variability can benefit from the development of new methods 11, 83, 136, including the possibility of using multimodal imaging to map the biological pathways that mediate individual differences in behaviour 129, 137.

Alt-text: Box 3

**Figure 2:**
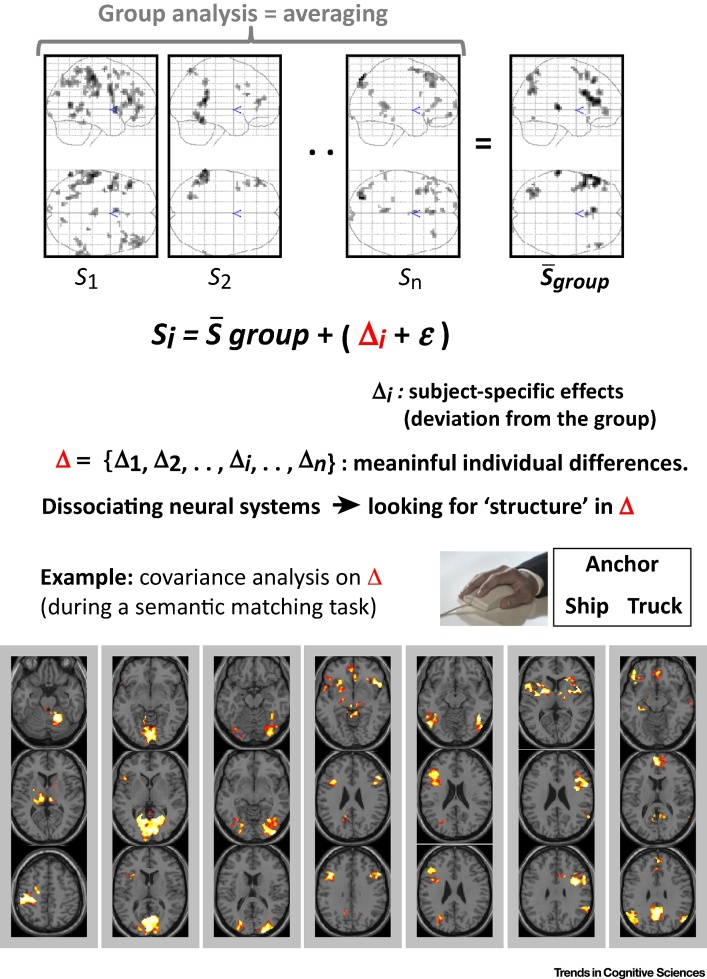
Segregation of Networks with Across-Subject Covariance Analyses. The figure illustrates the use of covariance analysis to segregate different networks associated with different strategies. Basically, if different personal biases for particular cognitive strategies exist, they can be distinguished from random errors by looking at similarity across brain regions in the between-subject variance. Top: different neuronal systems that can sustain the same task are dissociated using (clustering) algorithms that cluster together voxels if their associated deviations covary across subjects. Δ is the set of individual deviations from the population average. S_i_ is an activation summary of subject *i*, like an effect size, after collapsing the data across time or scans, and Δ_i_ codes potential biases including the personal bias of subject *i* to a particular cognitive strategy. Each Δ_i_ is a whole-brain map (for subject *i*), and ε codes inconsistent (measurement) noise. For example, Δ (in its simplest form) can represent a set of residuals from a group (mean) analysis, or a set of eigenimages after running a principal components analysis. Bottom: some of the networks that were segregated for a semantic matching task using an unsupervised fuzzy clustering algorithm (illustrated as red-to-yellow clusters projected on anatomical axial slices). In this example, subjects were asked to indicate with a button press if visually presented words were semantically related or not. Voxels were clustered together if their associated deviations covaried across subjects. This clustering revealed many networks, including motor, visual, semantic, default mode and oculomotor networks. More details about this example can be found elsewhere [67].

Several approaches have been used to model or find structure in between-subject variability 11, 68, 83, 84, 85. The approach illustrated in Figure 2 defines each subject’s activation map as a sum of two but unknown quantities: the mean population effect plus a subject-specific effect defined as a deviation from the mean at each brain voxel. The deviations can then be characterised according to their size or **covariance** across subjects that can be used to segregate functional networks. Moreover, the interpretation of intersubject variability 2, 13, 62, 63, 67, 68, 82, 86, 87, 88, 89, 90 has been facilitated by the fact that intrasubject (i.e., intersession) variability is smaller than intersubject variability [91], making subject-specific activation maps relatively stable [8] over many repetitions. For instance, it has been shown that fMRI signal variation at different parts of the brain can be predicted by individual differences in visual short-term memory capacity [92], motivational state [93], performance changes after practice [9], learning aptitude [94], attention shifting efficiency [95], cognitive flexibility [96], and inhibitory efficiency during executive functions [97]. These results clearly demonstrate that meaningful signal exists in the between-subject variance 90, 98 and highlights the possibility that there might be multiple alternative neural systems that can each support the task (i.e., degeneracy).

One way to dissociate alternative neural systems for a given task is to model between subject variance in activation (across a set of voxels) as a mixture of different subgroups, with the goal of maximising similarity within groups at the same time as maximising differences between groups. This rationale has previously been adopted when studying intersubject variability in healthy subjects 68, 85 and patients [99]. For instance, using a probabilistic classification method on fMRI activation maps from neurologically normal individuals, it was possible to segregate four subgroups of subjects who used different neural systems to read aloud the same set of regularly spelled English words [68]. *Post hoc* comparison of demographics and behavioural data showed that the four different groups differed in subject age and reading strategy, suggesting that intersubject variability may have been driven by these variables.

Insight into between-subject variability in functional activation, when the task is held constant, can also be gained by analysing how activation in different regions covary with one another [2]. This draws a parallel with the field of correlational psychology that looks at associations between behavioural variables [14]: if two tasks A and B are determined by the same construct, then a subject’s performance of task A should predict his/her performance of task B; if no correlation exists, a potential dissociation is suggested [17]. When looking at brain imaging data, we can reverse the inference by holding the task constant and looking at (de)associations between regional brain activations in the across-subject dimension. In this context, covariance is used to decode meaningful variability in the patterns of activation between subjects based on the assumption that regions belonging to the same network will have comparable variations from subject to subject 56, 67. The underlying assumption is that subjects engage many functional networks when performing the task, but, because the networks are functionally segregated (and spatially distinct), the level of activation in one network is not necessarily correlated, across subjects, with the level of activation in the other networks. The different processing networks can then be segregated by classifying brain voxels or regions according to their similarity in activation across subjects. For instance, using an unsupervised data-driven clustering algorithm across 39 healthy subjects, it was possible to segregate different networks involved in semantic categorisation (Figure 2), including other hidden networks that were not identified by standard cognitive subtraction approaches [67].

Covariance analyses can also be conducted in a hypothesis-driven way to segregate networks of regions associated with different strategies. This is particularly useful when a given brain region is known to be associated with a particular cognitive strategy. That region can then serve as a seed region in a search over the whole brain for regions where activation covaries similarly across subjects with that in the seed region 2, 100. Regions that strongly correlate across subjects with the seed region can then be hypothesised to be part of the same subsystem and thus associated with the same cognitive strategy as the seed region 2, 101. For instance, hypothesis-driven covariance analysis shows how brain activation [2] as well as lateralisation [102] in different subregions of the occipito-temporal cortex covary with different frontal regions during word processing in skilled adult readers.

In summary, examining structure in the across-subject covariance can be used to group subjects together according to similarity in their activation patterns (within group) and dissimilarity (between groups). It can also be used to group regions of the same network together in order to identify how brain activation covaries during the recruitment of different strategies. Covariance can therefore be used to characterise normal and abnormal variability, which has important applications for studying neurological or psychiatric conditions 103, 104.

## Concluding Remarks

Variability in brain function is more than noise (Box 3). The time has come for new developments in algorithmic and data processing that can answer questions beyond central tendency measures. These new methods should be an important complement to standard group inferences. Understanding between-subject variance holds the key to a better understanding of function–structure–behaviour associations (see Outstanding Questions). It will help to dissociate alternative (degenerate) available pathways that can sustain a given cognitive skill. We have highlighted the potential implications of this endeavour for clinical applications, particularly for prognoses purposes. Namely, by segregating the different processing neural systems, for a given skill, it may lead to accurate predictions about recovery after brain damage depending on whether all or a subset of systems were damaged or spared. Last but not least, as hundreds of functional neuroimaging studies are conducted every year, collaborative databasing initiatives are needed, with a particular emphasis on data mining in the across-subject dimension.

Outstanding Questions What are the factors that impact upon the strategy selected by subjects to perform a given task?

How is switching between different cognitive strategies governed by top-down and bottom-up processing?

How does the size of between-subject variability in clinical populations compare to that in typical populations?

How can this knowledge be used to optimise the definition of cut-off scores for diagnostic use of neuroimaging data?

How does functional and structural variability between humans compare to that seen in animals?

What are the links between variability in functional connectivity and behaviour?

How can variability be modelled in a way that could increase the reliability of group inferences?

What will high spatial resolution data (e.g., layer-specific fMRI) tell us about intersubject variability?

How can information about intersubject variability in brain function be used to benefit the analysis of data from other modalities (e.g., the inverse problem in electroencephalography)?

## Glossary

**Aggregate statistics**: statistics that summarise a group of measurements or observations.
**Brain connectivity**: afferent and efferent connections that mediate interactions between brain regions. Connectivity can be anatomical (white matter tracts), functional (statistical associations), or effective (causal interactions).
**Brain rhythms**: oscillations in electrical, magnetic, hemodynamic, or other measurements that are generated by activity in neurons or the interaction between neurons. Rhythms can be characterised by their frequency, amplitude and phase.
**Central tendency**: tendency of random variables to cluster round the centre or average. The most common central tendency measure is the arithmetic mean.
**Cognitive strategy**: sequence of mental processes for accomplishing a given task.
**Cognitive style**: personal bias to a particular cognitive strategy, due to genetics or prior experience.
**Covariance**: measure of how two variables change together, irrespective of their means. It is calculated by taking the product of the deviations of two variables from their respective means.
**Degeneracy** (in neurobiology): ability of elements that are structurally different to perform the same function or yield the same output.
**Dynamical system**: system that changes from one state to another over the course of time. It depicts a system in which motion or transitions occur.
**Functional anatomy**: relationship between brain anatomy and function. It defines brain regions using functional landmarks instead of purely structural landmarks.
**Lateralisation**: relative difference between the left and right hemisphere that results from the asymmetric processing of sensory and cognitive information.
**Measurement error**: random errors or existing bias that diminish the accuracy of the measurement. The presence of measurement error indicates that the results of sampling might not be duplicated across repetitions.
**Mesoscopic level**: intermediate level of representation between microscopic and macroscopic scales.
**Network**: set of interconnected brain regions that interact among themselves during the processing of a given task. Regions and connections are referred to as nodes and edges respectively.
**Noise** (in statistics): amount of unexplained variance in a sample. It is typically modelled as random data.
**Nuisance parameter**: any parameter of no interest that can influence the estimation of other parameters of interest and should therefore be accounted for in data analysis.
**Plasticity**: changes in brain anatomy or function that result from learning or environmental changes.
**Processing pathway**: set of brain connections and regions that can sustain processing of sensory, motor, or cognitive functions.
**Recovery**: improvement in the severity of a deficit over time, until the patient returns to the normal range.
**Reorganization**: ability of the brain to adapt and reorganise its structure–function relationships after insult.
**Task manipulation**: methodical way to implement stimulus or task changes in neuroimaging experiments.
